# INS/GPS/LiDAR Integrated Navigation System for Urban and Indoor Environments Using Hybrid Scan Matching Algorithm

**DOI:** 10.3390/s150923286

**Published:** 2015-09-15

**Authors:** Yanbin Gao, Shifei Liu, Mohamed M. Atia, Aboelmagd Noureldin

**Affiliations:** 1 College of Automation, Harbin Engineering University, 145 Nantong Street, Nangang District, Harbin 150001, China; E-Mail: gaoyanbin@hrbeu.edu.cn; 2 Department of Electrical and Computer Engineering, Royal Military College of Canada, P.O. Box 17000, Station Forces, Kingston, ON K7K 7B4, Canada; E-Mails: mohamed.maher.atia@gmail.com (M.M.A.); aboelmagd.noureldin@rmc.ca (A.N.)

**Keywords:** LiDAR, Scan Matching, Unmanned Ground Vehicle, Urban and Indoor Navigation

## Abstract

This paper takes advantage of the complementary characteristics of Global Positioning System (GPS) and Light Detection and Ranging (LiDAR) to provide periodic corrections to Inertial Navigation System (INS) alternatively in different environmental conditions. In open sky, where GPS signals are available and LiDAR measurements are sparse, GPS is integrated with INS. Meanwhile, in confined outdoor environments and indoors, where GPS is unreliable or unavailable and LiDAR measurements are rich, LiDAR replaces GPS to integrate with INS. This paper also proposes an innovative hybrid scan matching algorithm that combines the feature-based scan matching method and Iterative Closest Point (ICP) based scan matching method. The algorithm can work and transit between two modes depending on the number of matched line features over two scans, thus achieving efficiency and robustness concurrently. Two integration schemes of INS and LiDAR with hybrid scan matching algorithm are implemented and compared. Real experiments are performed on an Unmanned Ground Vehicle (UGV) for both outdoor and indoor environments. Experimental results show that the multi-sensor integrated system can remain sub-meter navigation accuracy during the whole trajectory.

## 1. Introduction

Navigation can be simply concluded to solve the problem of determining the time varying position and attitude of a moving object based on the equipped proprioceptive sensors (inertial sensors, odometer, *etc.*) and exteroceptive sensors (laser scanner, camera, *etc.*). The techniques of positioning can be roughly categorized into relative positioning and absolute positioning. Relative positioning is the process of calculating the current position by using a known initial position, and the estimated or known speed over elapsed time. Inertial Navigation System (INS), which is a self-contained system and is immune to interference, belongs to this category. Due to the advancements in microelectronics and micromachining technologies, inertial sensors have evolved from bulky and expensive mechanical design to compact and affordable grade. However, whether it be the traditional mechanical or the Micro-Electro-Mechanical System (MEMS)-based inertial sensors, they are subject to different level of systematic and stochastic errors. These errors will accumulate in the integration process and grow without bound in the long run. Absolute positioning is to make measurements with respect to given reference systems, including Global Positioning System (GPS), active beacons (like wireless local area network, Bluetooth, ultra-wideband, active Radio-frequency Identification (RFID), *etc.*), passive beacons (like passive RFID, Near Field Communication (NFC) [1,2]), and manmade or natural landmarks, *etc*. GPS can provide accurate position, velocity and time information all-day in all weather conditions anywhere on the earth when line of sight to at least four satellites are available. However, GPS is susceptible to signal jamming and outage in challenging environments like urban and indoor areas, where satellite signals are unreliable or unavailable. Therefore, both relative positioning and absolute positioning possess advantages and disadvantages, which makes the standalone positioning technique fail to achieve sustainable accuracy. This limitation can be solved by integrating relative positioning and absolute positioning techniques, like the integration of INS and GPS. Fruitful achievements have been gained in the different integration schemes of INS and GPS. However, urban and indoor environments still remain as challenges due to the unreliability and unavailability of GPS.

Another technique that is commonly used in positioning and mapping applications is Light Detection and Ranging (LiDAR). LiDAR can provide accurate bearing and range measurements about the objects in the environments at high frequency. These measurements at each sampling epoch is a sequence of scanned points representing the general contour of the environment observed at the location of the LiDAR, which is defined as a scan. Comparing the working environments of GPS and LiDAR, it is revealed that they work well under complementary environments [3]. Specifically, in open sky, there is less potential of satellites signals blockage for GPS, while LiDAR tends to have less measurements due to the limited scanning range. In this case, it is either difficult to extract features from LiDAR measurements or to successfully match scanned points to estimate pose. In urban area, where there are tall buildings around, GPS suffers from signal jamming and multipath, while LiDAR can detect the building walls which can be extracted as features in the LiDAR measurements. In indoor environments, GPS loses line of sight to satellites, while LiDAR measurements are rich and it is easy to extract features from them. Therefore, integrating GPS and LiDAR with INS can provide continuous corrections to INS. In this paper, this multi-sensor navigation system for both outdoor and indoor environments is introduced.

The organization of the paper is as follows: Section 2 provides a review of literatures related to LiDAR-based integrated navigation systems and scan matching methods. The quaternion-based INS mechanization is given in Section 3. Section 4 introduces the hybrid scan matching algorithm. Section 5 is the filter design. Section 6 presents the experimental results and analysis. Section 7 is the conclusion.

## 2. Literature Review

In the literature, LiDAR has been successfully applied on platforms like land vehicles, aerial vehicles and pedestrians to implement autonomous navigation, localization and mapping. The integration of LiDAR with other sensors has been intensively studied, like the integration with inertial sensors in [4,5,6], the integration with INS and camera in [7,8], the integration with GPS in [9,10], and integration with INS and GPS in [3,11]. In most of the INS/LiDAR integration works, LiDAR is loosely coupled with INS, where the pose estimation from LiDAR and INS respectively are fused through Kalman Filter (KF) or Partial Filter (PF). While in [12,13], the tight integration of LiDAR and INS are reported, where extracted feature information is used in the filter as update.

Scan matching is a common technique that has been adopted to estimate pose change from LiDAR measurements. The scan matching method can be broadly classified into three different categories: feature-based scan matching, point-based scan matching and mathematical property-based scan matching [14]. Feature-based scan matching method transforms the raw LiDAR scan points into a more efficient representation. By tracking the feature parameters change or pose change oven two scans, the pose change of the vehicle can be derived. Some commonly used features are line segments [15,16,17,18], corners and jump edges [19,20], lane markers [21] and curvature functions [22]. The point-based scan matching method directly deals with the raw LiDAR scan points by searching and matching corresponding points between two scans. The Iterative Closest Point (ICP) algorithm [23,24] and its variants [25] are the most popular methods to solve the point-based scan matching problem, due to their simplicity and effectiveness. For the mathematical property-based scan matching method, it can be based on histogram [26], Hough transformation [27], normal distribution transform [28] or cross-correlation [29].

In our earlier works [13] and [30], we respectively implemented tightly coupled and loosely coupled INS and LiDAR integration with feature-based scan matching method for 2D indoor navigation. The feature-based scan matching method is efficient and accurate. However, it relies on the features in the environments. If the system is extended to outdoor or indoor unconstructed environments, it may fail to work properly. Besides, a loosely coupled INS and LiDAR integration with ICP-based scan matching method is presented in [31]. ICP-based scan matching method is accurate and independent of environment features. Efforts are made to accelerate this scan matching method. However, iterations to optimally align the scans are inevitable.

Different from existing works, a multi-sensor integrated navigation system for both outdoor and indoor environments with an innovative scan matching algorithm is proposed and implemented on an Unmanned Ground Vehicle (UGV). The main contributions of this paper are summarized as below:

- GPS and LiDAR are used as aiding systems to alternatively provide periodic corrections to INS in different environments. A quaternion-based error model is used to fuse multi-sensor information.
- An innovative hybrid scan matching algorithm that combines feature-based scan matching method with ICP-based scan matching method is proposed due to their complementary characteristics.
- Based on the proposed hybrid scan matching algorithm, both loosely coupled and tightly coupled INS and LiDAR integration are implemented and compared using real experimental data.

## 3. Quaternion-Based INS Mechanization

The INS mechanization involves different frames and their transformation. The navigation solutions are represented in navigation frame while inertial sensors measure angular velocity and acceleration of the vehicle directly in the body frame. The definitions of the frames used in the paper are listed in Table 1.

**Table 1:** Frames definitions.

Frames	Definition
Body frame	Origin: Vehicle center of mass.
	Y: Longitudinal (forward) direction.
	X: Transversal (lateral) direction.
	Z: Up vertical direction.
Navigation frame	Origin: Vehicle center of mass.
	Y: True north direction.
	X: East direction.
	Z: Up direction.

There are many formulations to represent the orientation of the vehicle such as Direct Cosine Matrix (DCM), Eular angles, and quaternion. In this work, the quaternion formulation is used due to its computational efficiency and lack of singularities. The quaternion vector is denoted as:

(1) $$Q={\left[\begin{array}{cccc} q_{0} & q_{1} & q_{2} & q_{3} \end{array}\right]}^{T}$$

The quaternion-based rotation matrix from body frame to navigation frame $C_{b}^{n}$ is given in Equation (2), where the superscript n means the navigation frame and the subscript b denotes the body frame.

(2) $$C_{b}^{n}=\left[\begin{array}{ccc} q_{0}^{2}+q_{1}^{2}-q_{2}^{2}-q_{3}^{2} & 2\left(q_{1}q_{2}-q_{0}q_{3}\right) & 2\left(q_{1}q_{3}+q_{0}q_{2}\right)\\ 2\left(q_{1}q_{2}+q_{0}q_{3}\right) & q_{0}^{2}-q_{1}^{2}+q_{2}^{2}-q_{3}^{2} & 2\left(q_{2}q_{3}-q_{0}q_{1}\right)\\ 2\left(q_{1}q_{3}-q_{0}q_{2}\right) & 2\left(q_{2}q_{3}+q_{0}q_{1}\right) & q_{0}^{2}-q_{1}^{2}-q_{2}^{2}+q_{3}^{2} \end{array}\right]$$

### 3.1. Position Mechanization Equations

The position vector p is expressed in geodetic coordinate in the Earth-centered Earth-fixed (ECEF) frame. The position vector is given as:

(3) $$p=\left[\begin{array}{ccc} \varphi & \lambda & h \end{array}\right]$$

where φ is the latitude, λ is the longitude, h is the altitude. As the vehicle moves, the rate of change in position can be expressed as below:

(4) $$\dot{\varphi }=\frac{v_{n}}{R_{M}+h}$$

(5) $$\dot{\lambda }=\frac{v_{e}}{(R_{N}+h)cos\varphi }$$

(6) $$\dot{h}=v_{u}$$

where $v_{e}$, $v_{n}$ and $v_{u}$ are the velocity components in the east, north and up directions respectively. $R_{M}$ is the meridian radius of the ellipsoid while $R_{N}$ is the normal radius of the ellipsoid.

### 3.2. Velocity Mechanization Equations

The measurements of the three-axis accelerometer are the vehicle specific force in the body frame. It should be transformed to navigation frame first, and then integrates to derive vehicle velocity in the navigation frame. To transform the specific force from body frame to navigation frame, the quaternion-based rotation matrix is used. The transformation is given by Equation (7):

(7) $$f^{n}=C_{b}^{n}f^{b}$$

where $f^{b}={\left[\begin{array}{ccc} f_{x} & f_{y} & f_{z} \end{array}\right]}^{T}$ is specific force vector in body frame, and $f^{n}={\left[\begin{array}{ccc} f_{e} & f_{n} & f_{u} \end{array}\right]}^{T}$ is acceleration components in the navigation frame. However, the acceleration derived from Equation (7) cannot be used directly due to the following effects:

(1) Earth rotation rate $\omega_{ie}$. The earth rotation rate in the navigation frame is:

(8) $$\omega_{ie}^{n}={\left[\begin{array}{ccc} 0 & \omega_{ie}\cos\varphi & \omega_{ie}\sin\varphi \end{array}\right]}^{T}$$

(2) Angular velocity caused by the change of orientation of the navigation frame with respect to the earth $\omega_{en}^{n}$, which is also called transportation rate. It can be expresses as:

(9) $$\omega_{en}^{n}={\left[\begin{array}{ccc} -\frac{v_{n}}{R_{M}+h} & \frac{v_{e}}{R_{N}+h} & \frac{v_{e}\tan\varphi }{R_{N}+h} \end{array}\right]}^{T}$$

(3) The earth’s gravity field g. The gravity field vector in the navigation frame is:

(10) $$g={\left[\begin{array}{ccc} 0 & 0 & -g \end{array}\right]}^{T}$$

These factors need to be compensated for in $f^{n}$. The rate of change in velocity is given as:

(11) $$\dot{v}=f^{n}-\left(2\omega_{ie}^{n}+\omega_{en}^{n}\right)\times v+g$$

where $v={\left[\begin{array}{ccc} v_{e} & v_{n} & v_{u} \end{array}\right]}^{T}$ is velocity vector in the navigation frame, $\left(2\omega_{ie}^{n}+\omega_{en}^{n}\right)\times$ is the skew-symmetric matrix of $\left(2\omega_{ie}^{n}+\omega_{en}^{n}\right)$. Due to the high noise level of the MEMS grade inertial sensors, the earth rotation rate cannot be detected. Furthermore, when the vehicle moves in low speed, the transportation rate is negligible. Therefore, the simplified rate of change in velocity can be rewritten as:

(12) $$\dot{v}=f^{n}+g=C_{b}^{n}f^{b}+g$$

### 3.3. Attitude Mechanization Equations

The differential equation of the quaternion is [32]:

(13) $$\dot{Q}=\frac{1}{2}\left[\begin{array}{cc} 0 & -\omega_{nb}^{b}\\ \omega_{nb}^{b} & -\left(\omega_{nb}^{b}\right)\times \end{array}\right]Q$$

where $\omega_{nb}^{b}$ is the angular velocity of the body frame with respect to the navigation frame represented in the body frame. It is given in Equation (14). $\omega_{ib}^{b}={\left[\begin{array}{ccc} \omega_{x} & \omega_{y} & \omega_{z} \end{array}\right]}^{T}$ is the gyroscope measurement in the body frame, representing the angular velocity of the body frame with respect to the inertial frame.

(14) $$\omega_{nb}^{b}=\omega_{ib}^{b}-C_{n}^{b}\left(\omega_{ie}^{n}+\omega_{en}^{n}\right)$$

However, since the earth rotation rate and the transportation rate are negligible, Equation (14) can be simplified as:

(15) $$\omega_{nb}^{b}\approx \omega_{ib}^{b}$$

By implementing matrix multiplication in Equation (13), it can be rewritten as:

(16) $$\begin{array}{l} {\dot{q}}_{0}=\ldots (-q_{1}\omega_{x}-q_{2}\omega_{y}-q_{3}\omega_{z})\\ {\dot{q}}_{1}=\ldots (q_{0}\omega_{x}-q_{3}\omega_{y}+q_{2}\omega_{z})\\ {\dot{q}}_{2}=\ldots (q_{3}\omega_{x}+q_{0}\omega_{y}-q_{1}\omega_{z})\\ {\dot{q}}_{3}=\ldots (-q_{3}\omega_{x}+q_{1}\omega_{y}+q_{0}\omega_{z}) \end{array}$$

After updating the quaternion using Equation (16), the quaternion-based rotation matrix is also updated. The attitude of the UGV can be computed based on the relationship between attitude angles and the quaternion-based rotation matrix. The formulations of pitch, roll and azimuth are given as:

(17) $$\begin{array}{l} p=\sin^{-1}\left(C_{b}^{n}(3,2)\right)\\ r=\tan^{-1}\left(\frac{-C_{b}^{n}(3,1)}{C_{b}^{n}(3,3)}\right)\\ A=\tan^{-1}\left(\frac{-C_{b}^{n}(1,2)}{C_{b}^{n}(2,2)}\right) \end{array}$$

## 4. Hybrid Scan Matching Algorithm

### 4.1. Feature-Based Scan Matching Method

**Figure 1:**
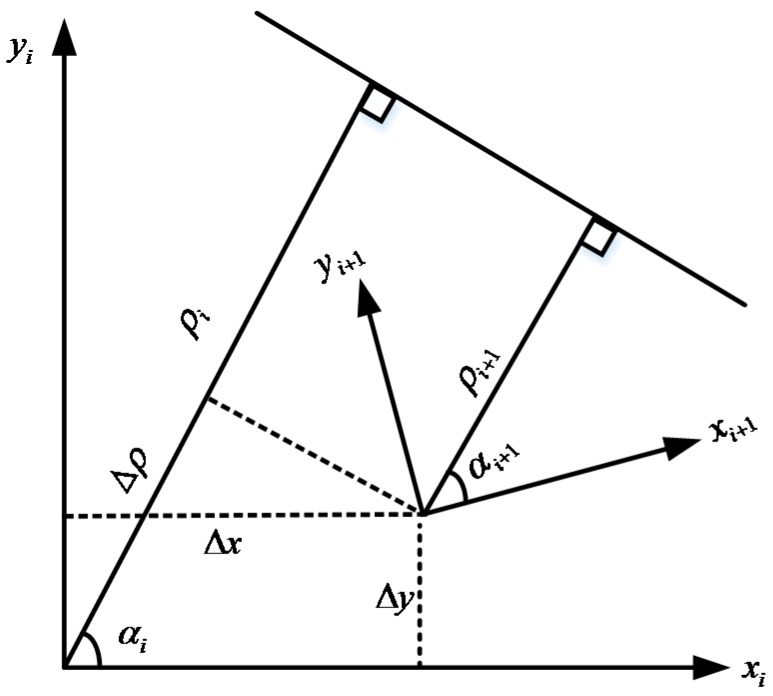
The vehicle pose change over two consecutive scans.

In this paper, the line feature is used due to its richness in indoor environments. The line feature is characterized by two parameters: the perpendicular distance from the origin of the vehicle body frame to the extracted line ρ, and the angle between the *x*-axis of the body frame and the norm of the extracted line α as shown in Figure 1. If the estimated vehicle relative position and azimuth changes Δx, Δy, and ΔA are used as LiDAR updates in the filter, it is defined as loosely coupled. In this case, at least two non-collinear line features have to be matched over two scans to accurately estimate relative position change. In the other integration scheme, change of the perpendicular distance from LiDAR to the line feature over two scans Δρ, and azimuth change ΔA are used as LiDAR updates, which is defined as tightly coupled. In this case, at least one line feature is required to be matched over two scans. Therefore, INS/LiDAR integration with line feature-based scan matching method cannot work when no line features are successfully matched over two scans.

Figure 1 demonstrates the same line feature in the body frame at epoch i and i+1. The relative position changes along both axes are defined as Δx and Δy, while the azimuth change is defined as ΔA. The change of the perpendicular distance from LiDAR to the line feature over two scans Δρ is connected with the vehicle relative position changes Δx and Δy in the following format:

(18) $$\Delta \rho =\rho_{i}-\rho_{i+1}=\Delta x\cos(\alpha_{i})+\Delta y\sin(\alpha_{i})$$

The relation between vehicle azimuth change and line feature angle change is straightforward, which can be represented as:

(19) $$\Delta A=\alpha_{i+1}-\alpha_{i}$$

### 4.2. Iterative Closest Point (ICP) Scan Matching Method

Given the current scan and the reference scan, the ICP-based scan matching method is described as follows:

(1) Inertial sensors provide initial rotation and translation;
(2) Transform the current scan using the current rotation and translation;
(3) For each point in the transformed current scan, find its two closest corresponding points in the reference scan;
(4) Minimize the sum of the square distance from point in the transformed current scan to the line segment containing the two closest corresponding points.
(5) Check whether the convergence is reached. If so, the algorithm will continue to process the next new scan. Otherwise, it will return to step two to search new correspondences again and repeat the procedures.

Although the point-to-line distance metric has quadratic convergence with a good initial rotation and translation guess, the correspondences search and the iteration are still time-consuming, which limits its ability for real-time application.

### 4.3. Hybrid Scan Matching Algorithm

From the description above, the featured-based scan matching method works accurately and efficiently in feature rich environments. However, it heavily relies on the features in the environments and has limited ability to cope with unstructured or cluttered environments. The ICP-based scan matching method can perform accurately and robustly in more general environments. However, it requires time-consuming correspondences search and iterations to optimally align the scans. It is easy to find out that the advantages and disadvantages of the two scan matching methods are complementary. Therefore, the hybrid scan matching algorithm is proposed to combine the two scan matching methods to benefit from the advantages and avoid the disadvantages. Specifically, the hybrid scan matching method will work in the default feature-based scan matching mode. However, when less than two non-collinear line features (for loosely coupled system) or no lines (for tightly coupled system) are matched over two scans, the ICP-based scan matching will be activated, and the hybrid scan matching algorithm will transit to ICP-based scan matching mode. In this way, the hybrid scan matching algorithm can achieve efficiency and robustness concurrently.

## 5. Filter Design

**Figure 2:**
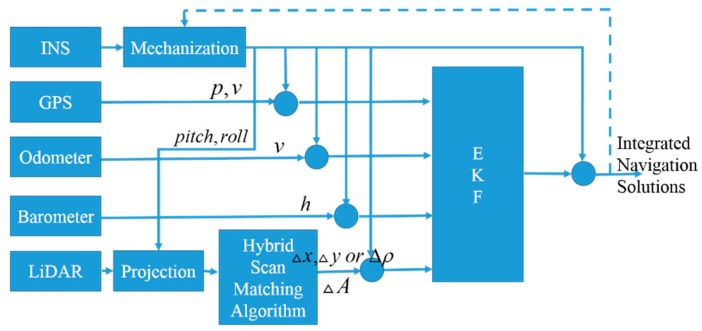
The block diagram of the multi-sensor integrated navigation system.

Information from multi-sensor are fused through an Extended Kalman Filter (EKF). The system model and measurement model are described in the following subsections. The block diagram of the multi-sensor integrated navigation system is shown in Figure 2.

### 5.1. System Model

In the proposed system, the error state vector for the EKF is defined as:

$$\delta x={\left[\begin{array}{cccc} \delta p & \delta v & \delta q & \begin{array}{cccc} \delta b_{\omega } & \delta b_{f} & \delta \Delta x & \begin{array}{cc} \delta \Delta y & \delta \Delta A \end{array} \end{array} \end{array}\right]}^{T}$$

where the elements in δx are error in position δp, error in velocity δv, error in quaternion δq, error in gyroscope bias $\delta b_{\omega }$, error in accelerometer biases $\delta b_{f}$, error in relative position changes δΔx, δΔy, and error in azimuth change δΔA respectively. $\delta b_{\omega }$ and $\delta b_{f}$ are modelled as first-order Gauss-Markov process, which is given in the following general form:

(20) $$\dot{x}\left(t\right)=-\beta x\left(t\right)+\sqrt{2\sigma^{2}\beta }w\left(t\right)$$

where σ is the standard deviation, β is the reciprocal of the time constant of the autocorrelation function of x(t) , and w(t) is zero-mean Gaussian noise. By applying Taylor expansion to INS position and velocity mechanization equations, quaternion differential equations, relative position changes and azimuth change equations, the linearized system error model is given as:

(21) $$\delta \dot{x}=F\delta x+Gw$$

where the transition matrix F can be written as [33]:

(22) $$F=\left[\begin{array}{cccccccc} F_{pp} & F_{pv} & 0_{3\times 4} & 0_{3\times 3} & 0_{3\times 3} & 0_{3\times 1} & 0_{3\times 1} & 0_{3\times 1}\\ 0_{3\times 3} & 0_{3\times 3} & F_{vq} & 0_{3\times 3} & -C_{n}^{b} & 0_{3\times 1} & 0_{3\times 1} & 0_{3\times 1}\\ 0_{4\times 3} & 0_{4\times 3} & \frac{1}{2}F_{qq} & F_{qb\omega } & 0_{4\times 3} & 0_{4\times 1} & 0_{4\times 1} & 0_{4\times 1}\\ 0_{3\times 3} & 0_{3\times 3} & 0_{3\times 4} & F_{b\omega } & 0_{3\times 3} & 0_{3\times 1} & 0_{3\times 1} & 0_{3\times 1}\\ 0_{3\times 3} & 0_{3\times 3} & 0_{3\times 4} & 0_{3\times 3} & F_{bf} & 0_{3\times 1} & 0_{3\times 1} & 0_{3\times 1}\\ 0_{1\times 3} & F_{\Delta xv} & 0_{1\times 4} & 0_{1\times 3} & 0_{1\times 3} & 0 & 0 & F_{\Delta x\Delta A}\\ 0_{1\times 3} & F_{\Delta yv} & 0_{1\times 4} & 0_{1\times 3} & 0_{1\times 3} & 0 & 0 & F_{\Delta y\Delta A}\\ 0_{1\times 3} & 0_{1\times 3} & 0_{1\times 4} & F_{\Delta Ab\omega } & 0_{1\times 3} & 0 & 0 & 0 \end{array}\right]$$

In the derivation of F matrix, the terms that are divided by the square of the earth radius are ignored. The elements in F matrix are given as below:

$$\begin{array}{ll} F_{pp}=\left[\begin{array}{ccc} 0 & 0 & 0\\ v_{e}\tan\varphi /\left(\left(R_{N}+h\right)\cos\varphi \right) & 0 & 0\\ 0 & 0 & 0 \end{array}\right], & F_{pv}=\left[\begin{array}{ccc} 0 & 1/(R_{M}+h) & 0\\ 1/(\left(R_{N}+h\right)\cos\varphi ) & 0 & 0\\ 0 & 0 & 1 \end{array}\right],\\ F_{vq}=\left[\begin{array}{cccc} F_{vq1} & F_{vq2} & F_{vq3} & F_{vq4}\\ -F_{vq4} & -F_{vq3} & F_{vq2} & F_{vq1}\\ F_{vq3} & -F_{vq4} & -F_{vq1} & F_{vq2} \end{array}\right], \end{array}$$

where $F_{vq1}=2\left(q_{0}(f_{x}-b_{fx})-q_{3}(f_{y}-b_{fy})+q_{2}(f_{z}-b_{fz})\right)$,

$$\begin{array}{l} F_{vq2}=2\left(q_{1}(f_{x}-b_{fx})+q_{2}(f_{y}-b_{fy})+q_{3}(f_{z}-b_{fz})\right),\\ F_{vq3}=2\left(-q_{2}(f_{x}-b_{fx})+q_{1}(f_{y}-b_{fy})+q_{0}(f_{z}-b_{fz})\right),\\ F_{vq4}=2\left(-q_{3}(f_{x}-b_{fx})-q_{0}(f_{y}-b_{fy})+q_{1}(f_{z}-b_{fz})\right),\\ F_{qq}=\left[\begin{array}{cccc} 0 & -(\omega_{x}-b_{\omega x}) & -(\omega_{y}-b_{\omega y}) & -(\omega_{z}-b_{\omega z})\\ \omega_{x}-b_{\omega x} & 0 & \omega_{z}-b_{\omega z} & -(\omega_{y}-b_{\omega y})\\ \omega_{y}-b_{\omega y} & -(\omega_{z}-b_{\omega z}) & 0 & \omega_{x}-b_{\omega x}\\ \omega_{z}-b_{\omega z} & \omega_{y}-b_{\omega y} & -(\omega_{x}-b_{\omega x}) & 0 \end{array}\right],F_{qb\omega }=-\frac{1}{2}\left[\begin{array}{ccc} -q_{1} & -q_{2} & -q_{3}\\ q_{0} & -q_{3} & q_{2}\\ q_{3} & q_{0} & -q_{1}\\ -q_{2} & q_{1} & q_{0} \end{array}\right], \end{array}$$

$F_{b\omega }=diag\left(-\beta_{\omega x},-\beta_{\omega y},-\beta_{\omega z}\right)$, $F_{bf}=diag\left(-\beta_{fx},-\beta_{fy},-\beta_{fz}\right)$, where $\beta_{\omega x},\beta_{\omega y},\beta_{\omega z}$ and $\beta_{fx},\beta_{fy},\beta_{fz}$ are the reciprocal of the time constant of the random process associated with the gyroscope bias and accelerometer bias respectively.

$$\begin{array}{l} F_{\Delta xv}=\frac{\cos p\sin\Delta A}{v}\left[\begin{array}{ccc} v_{e} & v_{n} & v_{u} \end{array}\right],F_{\Delta yv}=\frac{\cos p\cos\Delta A}{v}\left[\begin{array}{ccc} v_{e} & v_{n} & v_{u} \end{array}\right],F_{\Delta x\Delta A}=v\cos p\cos\Delta A,\\ F_{\Delta y\Delta A}=-v\cos p\sin\Delta A,F_{\Delta Ab\omega }=\left[\begin{array}{ccc} \cos p\sin r & -\sin p & -\cos p\cos r \end{array}\right]. \end{array}$$

where $v=\sqrt{v_{e}^{2}+v_{n}^{2}+v_{u}^{2}}$ is vehicle velocity. The process noise vector contains the noises in gyroscope measurements, noises in accelerometer measurements, and the Gaussian noises associated in the first order Gauss-Markov process of gyroscope bias and accelerometer bias. The non-zeros elements in the noise coupling matrix G are:

$$\begin{array}{l} G\left(4:6,4:6\right)=C_{n}^{b},G\left(7:10,1:3\right)=-F_{qb\omega },G\left(19,1:3\right)=-F_{\Delta Ab\omega },\\ G\left(11:13,7:9\right)=diag(\sqrt{2\sigma_{\omega x}^{2}\beta_{\omega x}},\sqrt{2\sigma_{\omega y}^{2}\beta_{\omega y}},\sqrt{2\sigma_{\omega z}^{2}\beta_{\omega z}}), \end{array}$$

$G\left(14:16,10:12\right)=diag(\sqrt{2\sigma_{fx}^{2}\beta_{fx}},\sqrt{2\sigma_{fy}^{2}\beta_{fy}},\sqrt{2\sigma_{fz}^{2}\beta_{fz}})$, where $\sigma_{\omega x},\sigma_{\omega y},\sigma_{\omega z}$ and $\sigma_{fx},\sigma_{fy},\sigma_{fz}$ are the standard deviation of the gyroscope bias and accelerometer bias respectively.

### 5.2. Measurement Model

#### 5.2.1. GPS Measurements

In outdoor environments where GPS signals are available, position and velocity solutions from GPS are integrated with INS at the rate of 1 Hz. The measurement model for INS/GPS loosely coupled scheme is:

(23) $$\left[\begin{array}{c} p_{gps}-p_{ins}\\ v_{gps}-v_{ins} \end{array}\right]=I_{6\times 6}\left[\begin{array}{c} \delta p\\ \delta v \end{array}\right]$$

where the subscript means the corresponding system measurements.

#### 5.2.2. LiDAR Measurements

The relative position and azimuth changes from LiDAR and INS respectively are integrated at the rate of 5 Hz. If the estimated vehicle relative position and azimuth changes Δx, Δy, and ΔA are used as LiDAR measurements, the measurement model can be given in Equation (24). This measurement model is used when INS and LiDAR are loosely coupled with feature-based scan matching method or ICP-based scan matching method.

(24) $$\left[\begin{array}{c} \Delta x_{lidar}-\Delta x_{ins}\\ \Delta y_{lidar}-\Delta y_{ins}\\ \Delta A_{lidar}-\Delta A_{ins} \end{array}\right]=I_{3\times 3}\left[\begin{array}{c} \delta \Delta x\\ \delta \Delta y\\ \delta \Delta A \end{array}\right]$$

where $\Delta x_{ins}$, $\Delta y_{ins}$ and $\Delta A_{ins}$ are given as below:

(25) $$\Delta x_{ins}=v\cos p\sin\Delta A\cdot T$$

(26) $$\Delta y_{ins}=v\cos p\cos\Delta A\cdot T$$

(27) $$\Delta A_{ins}=\omega_{z}^{l}T$$

where T is sampling interval, and $\omega_{z}^{l}$ is the angular velocity along the vertical axis projected in the horizontal plane, where all LiDAR scanned points are projected into before line feature extraction and scan matching procedures. It can be written as:

(28) $$\omega_{z}^{l}=-\omega_{x}\cos p\sin r+\omega_{y}\sin p+\omega_{z}\cos p\cos r$$

If change of the perpendicular distance from LiDAR to the line feature over two scans Δρ, and azimuth change ΔA are used as LiDAR measurements, the measurement model can be given in Equation (29). This measurement model is used when INS and LiDAR are tightly coupled with feature-based scan matching method.

(29) $$\left[\begin{array}{c} \Delta \rho_{lidar,1}-\Delta \rho_{ins,1}\\ \ldots \\ \Delta \rho_{lidar,n}-\Delta \rho_{ins,n}\\ \Delta A_{lidar,1}-\Delta A_{ins,1}\\ \ldots \\ \Delta A_{lidar,n}-\Delta A_{ins,n} \end{array}\right]=\left[\begin{array}{cc} \begin{array}{c} \begin{array}{cc} \cos\alpha_{1} & \sin\alpha_{1} \end{array}\\ \ldots \\ \begin{array}{cc} \cos\alpha_{n} & \sin\alpha_{n} \end{array} \end{array} & 0_{n\times 1}\\ 0_{n\times 2} & I_{n\times 1} \end{array}\right]\left[\begin{array}{c} \delta \Delta x\\ \delta \Delta y\\ \delta \Delta A \end{array}\right]$$

where n is the number of line features that has been matched over two scans, and n≥1 should holds. $\Delta \rho_{ins,j}$ represents the perpendicular distance change of the jth matched line feature pair over two scans. It has the following format:

(30) $$\Delta \rho_{ins,j}=\Delta x_{ins}\cos\left(\alpha_{j}\right)+\Delta y_{ins}\sin\left(\alpha_{j}\right)$$

#### 5.2.3. Odometer and Barometer Measurements

The velocity from odometer and height information from barometer are fused with INS velocity and altitude estimation at the rate of 10 Hz. The measurement model is written as below:

(31) $$\left[\begin{array}{c} v_{odo}-v_{ins}\\ h_{bar}-h_{ins} \end{array}\right]=I_{4\times 4}\left[\begin{array}{c} \delta v\\ \delta h \end{array}\right]$$

## 6. Experimental Results and Analysis

**Figure 3:**
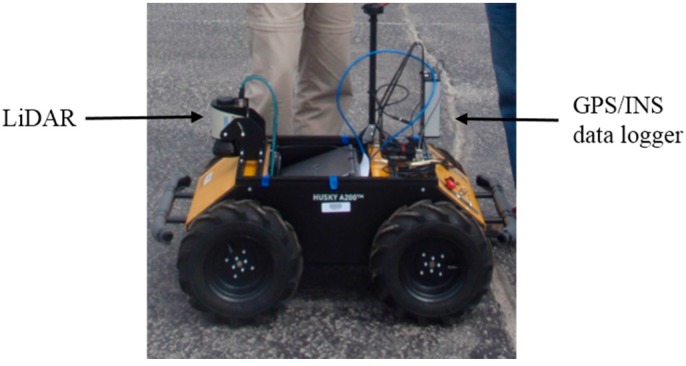
Experiment platform: Husky A200.

The UGV used in the experiment is called Husky A200 from Clearpath Robotics Inc. (Kitchener, ON, Canada) as shown in Figure 3. This UGV is wirelessly controlled and the platform is equipped with a quadrature encoder, a SICK laser scanner LMS111 [34] and a GPS/INS data logger that includes a GNSS receiver (u-blox LEA-6T), an IMU from VTI Technologies Inc., and a barometer from Measurements Specialties.

The experiment starts outdoors on a square without building or foliage blockage. Then the UGV travels from outdoor into the building through four ramps. For the rest of the experiment, the UGV moves along the corridor clockwise for one loop and stops in front of the elevator where it starts the motion indoors. While moving in the corridor, the UGV enters a copy room, a classroom and a lab room respectively. The length of the trajectory that the UGV travels is around 440 m and the travelling time is around 16 min. GPS is integrated with INS/odometer/barometer in the beginning of the experiment outdoor for 234 s before the GPS solutions become unreliable. Then, LiDAR replaces GPS to integrate with INS/odometer/barometer in the ramps part and indoor environments.

**Figure 4:**
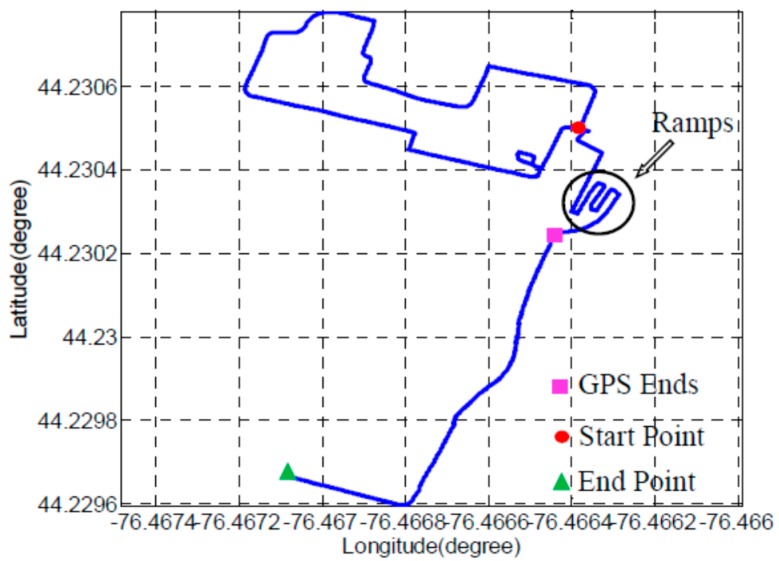
The generated trajectory from tightly coupled navigation system.

Two integration scheme of INS and LiDAR with hybrid scan matching algorithm are implemented and compared. The generated trajectories are very close and only the trajectory from tightly coupled navigation system is presented in Figure 4.

**Figure 5:**
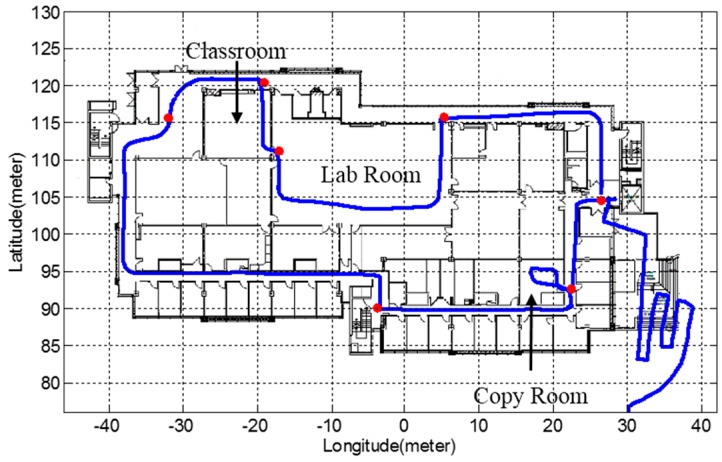
Indoor part trajectory.

For better presentation, the trajectory after GPS ends has been rotated counter clockwise by an angle of 17 degrees. For the indoor part, the trajectory is plotted on the map of the building. While traveling indoor, the UGV stops at seven points for at least 10 s to generate reference locations, which are defined as waypoints. The LiDAR-aided part trajectory, the rooms that UGV enters, and seven waypoints are illustrated in Figure 5.

During the LiDAR-aided navigation period, the filter implements 3527 times LiDAR measurement updates. For loosely coupled INS/LiDAR system with hybrid scan matching algorithm, the hybrid scan matching algorithm works in line feature-based scan matching mode for most of the time, and ICP-based scan matching mode is activated for 135 times. Among them, 105 times happen outside on the ramps and 30 times occur mostly in the copy room and classroom. This is reasonable since the railings of the ramps are made of vertical metal bars, and the laser beams can go through the space between the bars and does not reflect back. This will cause discontinuous short line segments. The line segments shorter than a threshold will be discarded in the line extraction process. Therefore, in unstructured environments like ramps and rooms with cluttered objects, it is difficult to extract and match at least two non-collinear line features.

However, for tightly coupled INS/LiDAR system with hybrid scan matching algorithm, ICP-based scan matching mode is activated for only 6 times and all of them happen on the ramps. This means for 99.83% of the trajectory, at least one line feature can be matched over two scans. The comparison of the two integration schemes of INS and LiDAR with hybrid scan matching algorithm is shown in Table 2. It can be clearly seen from the table, while performing the hybrid scan matching algorithm, the line feature-based scan matching scheme plays a dominant role, which maintains the computational efficiency. The ICP-based scan matching mode fills the gaps when less than two non-collinear line features (for loosely couple) or no line features (for tightly coupled) are matched over two scans. This guarantees the accuracy and robustness of the LiDAR-derived measurement updates.

**Table 2:** Comparison of loosely coupled and tightly coupled INS/LiDAR systems.

Integration Schemes	Feature-Based Scan Matching Activated Times (Percentage)	ICP-Based Scan Matching Activated Times (Percentage)	
		Outdoor	Indoor
Loosely coupled system	3392 (96.17%)	105 (2.98%)	30 (0.85%)
Tightly coupled system	3521 (99.83%)	6 (0.17%)	0

Position errors are calculated at the waypoints to assess the accuracy of the integrated solutions after GPS ends for INS, INS/LiDAR loosely coupled and tightly coupled systems. The results are demonstrated in Table 3. For INS, the position solutions are derived based on dead reckoning. As UGV moves, the position error of INS standalone grows gradually and reaches 13.53 m in the end. This is unsatisfactory for accurate indoor navigation. In contrast, the integrated navigation systems (loosely coupled and tightly coupled) can remain an average sub-meter level accuracy.

**Table 3:** Position errors.

Localization Errors(m)	1	2	3	4	5	6	7	Average
INS	1.43	4.66	10.96	8.73	6.22	6.48	13.53	7.43
Loosely coupled system	0.18	0.69	0.62	0.60	0.46	0.73	0.60	0.55
Tightly coupled system	0.12	0.45	0.27	0.63	0.32	0.51	0.80	0.44

If we compare the loosely coupled system and tightly coupled system, it can be found that the position accuracy of the tightly coupled system is slightly higher than the loosely coupled system with shorter running time and less limitation on the environment structure. This makes the tightly coupled system preferable over the loosely coupled system.

**Figure 6:**
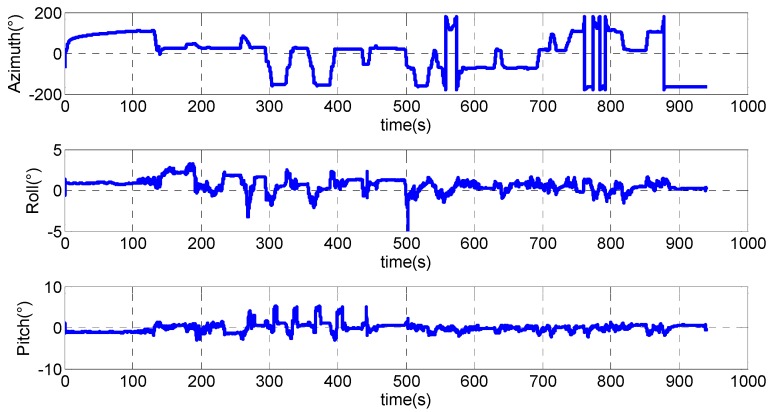
Attitude angles.

The attitude angles are shown in Figure 6. As can be seen from the figure, pitch and roll are relatively smaller when the UGV is stationary or in indoor environments. During the period of 300 s to 400 s, pitch grows from around zeros degree to around five degrees and then decreases to zeros degree again for four times. This corresponds to the four ramps with an inclination angle of five degrees.

## 7. Conclusions

In this paper, a multi-sensor integrated navigation system for both urban and indoor environments has been proposed. GPS and LiDAR are used as aiding systems to alternatively provide periodic corrections to INS in different environments since they work in complementary environments. Besides, due to the complementary properties of feature-based scan matching method and ICP-based scan matching method, the hybrid scan matching algorithm is proposed to combine the two scan matching methods to benefit from the advantages of each method. Two integration schemes of INS and LiDAR are implemented and compared. The tightly coupled system is preferable over the loosely coupled system when considering accuracy, running time and limitations on environments. Experimental results show that sub-meter navigation accuracy and accurate attitude angles estimation can be accomplished during the whole trajectory including outdoor and indoor environments.
